# Cardiac Sarcoidosis With Elevated Cardiac Troponin Mimicking Acute Myocardial Ischemia: A Case Report

**DOI:** 10.7759/cureus.35948

**Published:** 2023-03-09

**Authors:** Bruce C Casipit, Hussein Al-Sudani, Aman Amanullah

**Affiliations:** 1 Internal Medicine, Einstein Medical Center Philadelphia, Philadelphia, USA; 2 Internal Medicine, Einstein Medical Center Montgomery, East Norriton, USA; 3 Cardiology, Einstein Medical Center Philadelphia, Philadelphia, USA

**Keywords:** case report, cardiac magnetic resonance imaging, troponin elevation, sarcoidosis, cardiac sarcoidosis

## Abstract

Cardiac sarcoidosis (CS) is a disease entity with variable presentation causing significant morbidity and mortality. Concurrent signs of myocardial injury as evidenced by troponin elevation add to the complexity of an already challenging diagnosis. We present an unusual case of CS with elevated troponin I mimicking an acute ischemic cardiac event.

A 48-year-old female presented with a two-month history of presyncope.  Electrocardiogram showed a bifascicular block with concomitant significant troponin I elevation. Two-dimensional echocardiography showed new-onset left ventricular systolic dysfunction with an ejection fraction of 40-45%. A heparin drip was initiated for possible non-ST-elevation myocardial infarction. Coronary angiography showed no evidence of epicardial coronary artery disease but did show an anomalous right coronary artery; however, CT angiography did not reveal any significant stenosis. Further, the telemetry monitor captured intermittent complete atrioventricular blocks. Due to concerns for an infiltrative cardiac disease, a cardiac magnetic resonance was done showing findings consistent with possible CS.  CT scan of the chest showed no radiographic evidence of pulmonary sarcoidosis. Fluorodeoxyglucose-positron emission tomography scan showed findings of active inflammation in the myocardium consistent with possible CS. The patient was treated for clinical CS with systemic corticosteroids and methotrexate. Follow-up six weeks later showed clinical improvement of symptoms.

Our clinical case encompasses the unique variable presentation of CS including cardiac conduction abnormalities and left ventricular systolic dysfunction. Concomitant troponin I elevation can mimic myocardial ischemia, making the diagnosis more challenging. Treatment strategies aim to mitigate the long-term effects of CS on the heart; however, there is a paucity of data for appropriate pharmacological regimens.

## Introduction

Cardiac sarcoidosis (CS) is clinically manifested in approximately 5% of patients with systemic sarcoidosis [1,2]. CS has a variable presentation including new-onset cardiomyopathy or conduction abnormalities [3], which presents as a diagnostic challenge because a definitive diagnosis with endomyocardial biopsy is often not feasible and diagnostic yield is typically low [3]. Concomitant troponin elevation, which may be seen in the active inflammatory phase of CS, may be mistaken for acute myocardial infarction (AMI). This makes the diagnosis more challenging because concomitant AMI also presents with troponin elevation and possibly new-onset conduction abnormalities and cardiomyopathy. We present an unusual case of clinically diagnosed CS on imaging initially presenting with new-onset conduction abnormalities and systolic dysfunction with concomitant troponin elevation.

## Case presentation

A 48-year-old female with no significant past medical and family history presented to the hospital for a two-month history of presyncopal episodes characterized as almost passing out and occasionally associated with palpitations. Physical examination showed that she had a temperature of 37.2°C, heart rate of 84 beats/minute, respiratory rate of 16 breaths/minute, blood pressure of 129/81 mmHg, and SpO_2_ of 100% on room air. Her cardiopulmonary examination was unremarkable, with a regular rate and rhythm and no murmurs, and her lungs were clear to auscultation. Laboratory studies showed a troponin I elevation of 7.29 ng/mL (0-0.04 ng/mL). Electrolytes, renal, and liver function tests were unremarkable. The patient at that time denied any chest pain or shortness of breath.

**Figure 1:**
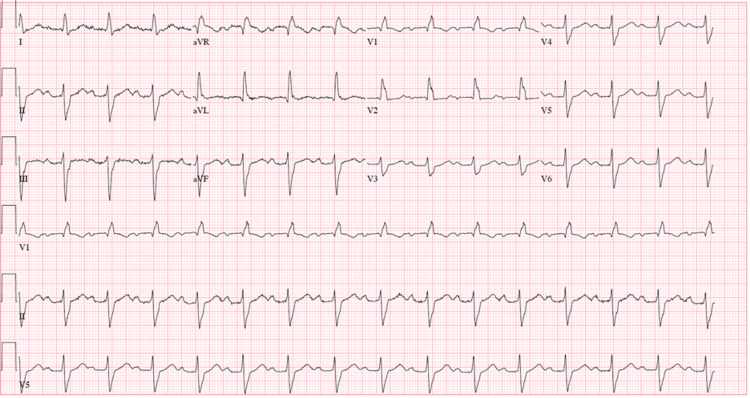
Electrocardiogram showing sinus rhythm with first-degree arteriovenous block, right bundle branch block, and left anterior fascicular block consistent with a bifascicular block.

An electrocardiogram (ECG) showed a right bundle branch block (RBBB) and left anterior fascicular block (LAFB) consistent with a bifascicular block (Figure 1).

Echocardiography showed a reduced left ventricular systolic function with an ejection fraction (EF) of 40-45% and severe hypokinesis of the basal to mid-inferoseptal walls (Video 1).

**Video 1:** Two-dimensional echocardiography apical four-chamber view showing reduced left ventricular systolic function with an ejection fraction of 40-45% and severe hypokinesis of the basal to mid-inferoseptal walls.

**Figure 2:**
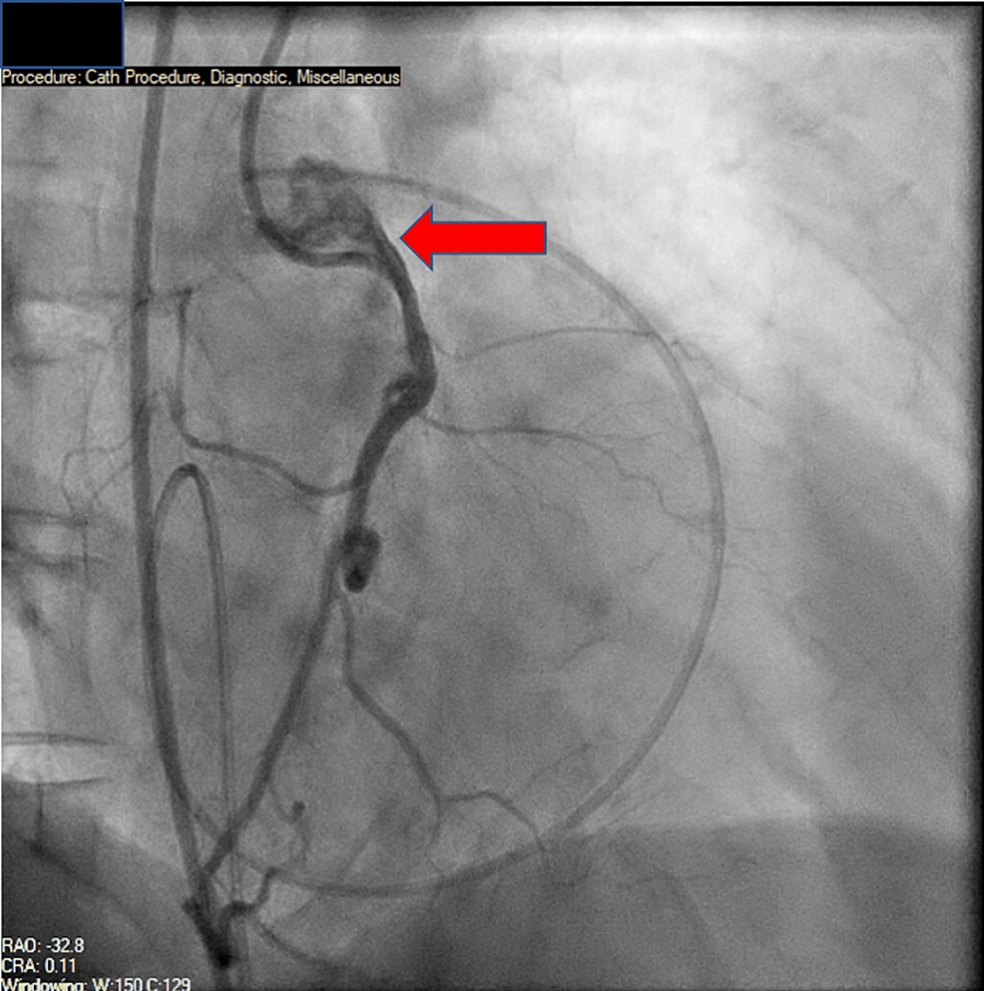
Coronary angiogram showing an anomalous right coronary artery arising from the left coronary cusp (red arrow).

Coronary angiography did not show any epicardial coronary artery disease but showed an anomalous right coronary artery (RCA) originating from above the left coronary sinus (Figure 2).

During this time, the patient was started on a heparin drip for non-ST-elevated myocardial infarction (NSTEMI) thought to be caused by hemodynamically significant stenosis of the anomalous RCA.

**Figure 3:**
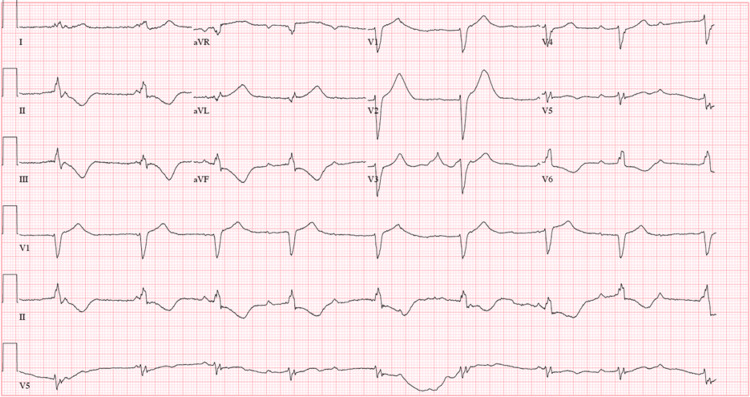
Electrocardiogram showing sinus rhythm with arteriovenous dissociation and wide QRS rhythm and left bundle branch block.

During the hospitalization, the patient developed episodes of complete heart block (Figure 3) with associated dizziness.

**Figure 4:**
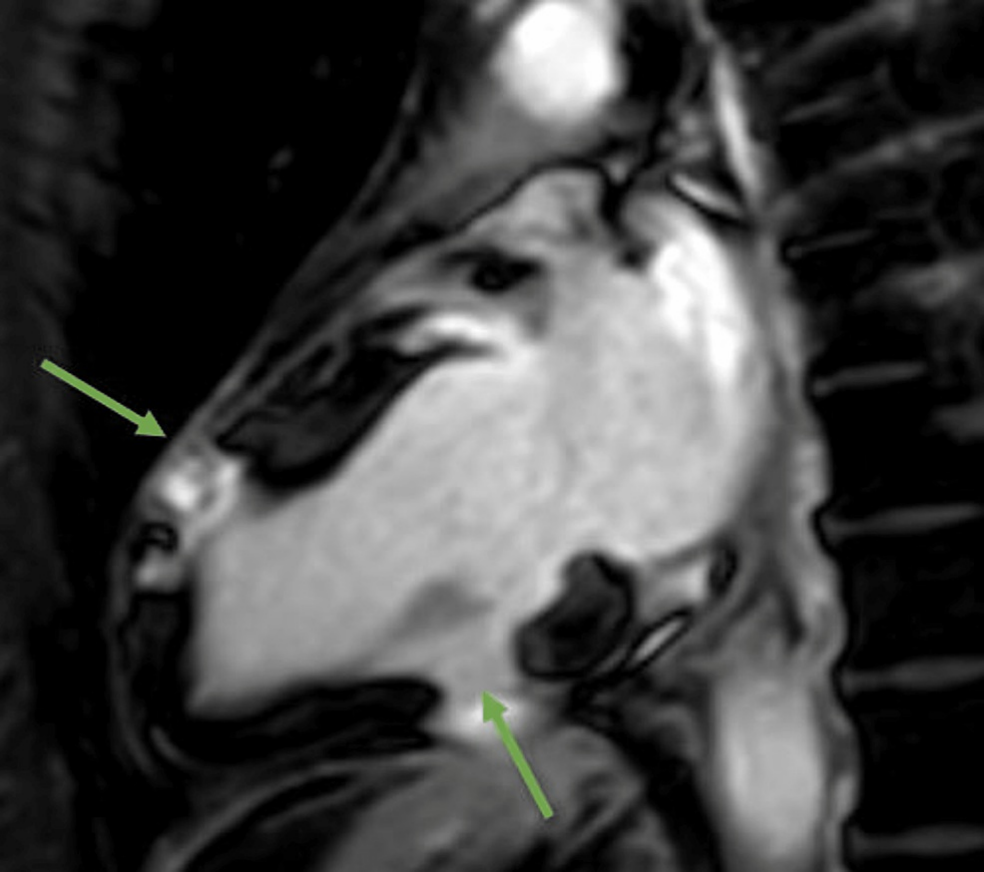
Phase-sensitive, inversion recovery sequence two-chamber view showing patchy (subendocardial and near-transmural) areas of late gadolinium enhancement in the inferior and inferoseptal wall of the left ventricle (green arrow) consistent with possible active inflammatory phase of cardiac sarcoidosis.

Because the degree of conduction abnormalities and left ventricular systolic dysfunction were out of proportion to the cardiac catheterization findings, a workup was done to assess for the presence of a cardiac infiltrative process. Cardiac magnetic resonance (CMR) imaging was done which showed several distinct areas of marked hypokinesis involving the basal septum, basal to mid-inferoseptal wall, and apical to mid-anteroseptal wall with a left ventricular EF of 42%. Further, there were constellations of findings including patchy subendocardial and near-transmural areas of late gadolinium enhancement (LGE) in the inferior and inferoseptal wall of the left ventricle which appeared to be partially hypoperfused and with associated focal hypokinesis and focal myocardial edema which was consistent with possible active inflammatory phase of CS (Figure 4).

The differential diagnosis of CS was made in the setting of conduction abnormalities and new-onset left ventricular dysfunction in the background of abnormal CMR findings. Other differential diagnoses that were considered at that time were myocarditis and pericarditis. However, it was deemed that myocarditis and pericarditis were less likely in this case given the progression of symptoms occurred over a period of time, which is usually the case in infiltrative diseases compared to a more acute onset seen in myocarditis and pericarditis; however, in some cases, these disease entities can present chronically in the setting of an underlying autoimmune condition which the patient did not have. Further, the patient did not complain of any chest pain at any point which is a typical finding in myocarditis and pericarditis. Finally, the patient did not have any viral prodrome or notable history of medication intake that would raise suspicion for myocarditis and pericarditis.

**Figure 5:**
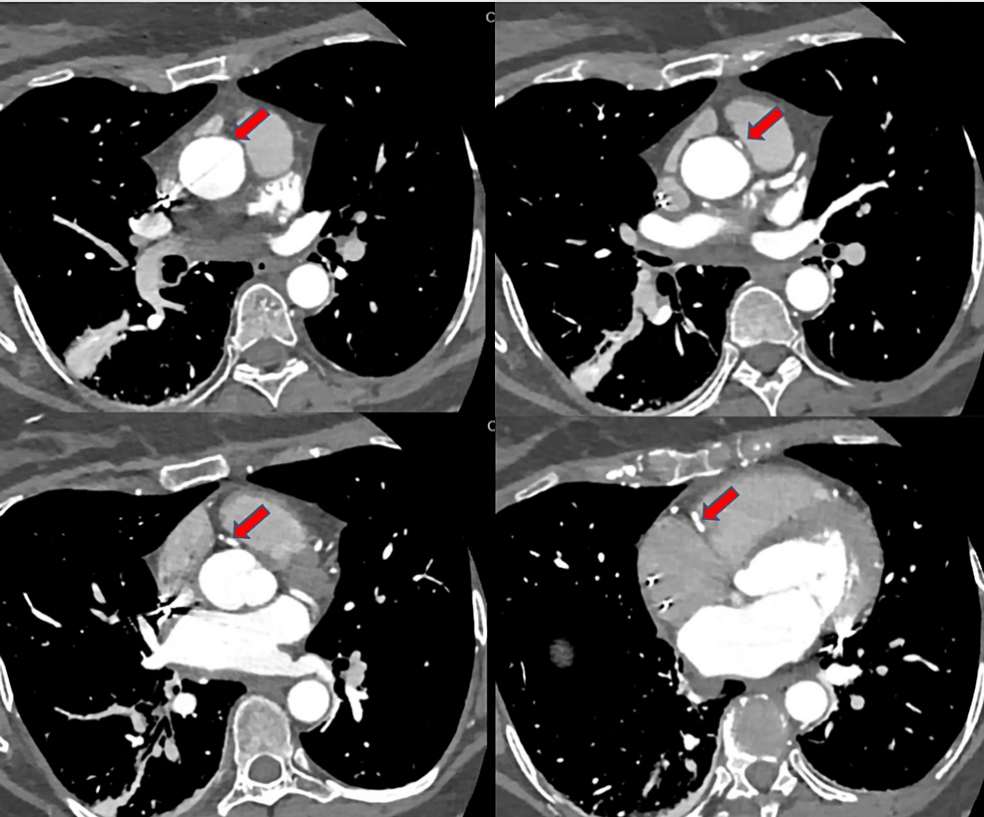
CT coronary angiogram showing an anomalous right coronary artery (red arrows) originating from the left anterolateral wall of the proximal ascending aorta above the sinotubular junction.

CT coronary angiogram was done to assess whether these findings were related to the anomalous RCA found on cardiac catheterization which showed no evidence of significant stenosis (Figure 5).

This further supported the diagnosis of presumptive CS because myocardial ischemia, which can also cause left ventricular systolic dysfunction and conduction abnormalities, was objectively ruled out. A biventricular defibrillator was implanted for the significant conduction abnormalities resulting in left ventricular dysfunction and concern for ventricular arrhythmias in the future.

Post-procedure, the patient developed ventricular tachycardia (VT) which was managed with overdrive pacing. A chest CT scan was done which did not reveal any bilateral hilar lymphadenopathy concerning for possible pulmonary sarcoidosis. Fluorodeoxyglucose-positron emission tomography (FDG-PET) scan showed findings of active inflammation in the myocardium consistent with possible CS.

The patient was started on oral prednisone 40 mg daily with taper for eight weeks and oral methotrexate 17.5 mg weekly. Interim history showed that there was no recurrence of the VT or conduction abnormalities. Just before discharge, troponin I went down to 2.1 ng/mL (0-0.04 ng/mL). A follow-up six weeks later showed clinical improvement of symptoms, and a repeat echocardiogram showed a left ventricular EF of 55%.

## Discussion

CS is clinically manifested in 5% of patients diagnosed with extracardiac sarcoidosis [1,2]. Clinical features of CS depend on the location, extent, and activity of the disease [2]. Variations of the clinical manifestations include conduction abnormalities, ventricular arrhythmias, sudden death, or new-onset or worsening heart failure [3].

Conduction abnormalities are the most common manifestations of CS with a prevalence of about 62%. Complete heart blocks and bundle branch blocks are seen in about 23-30% and 12-32%, respectively [3]. This was evident in our case in which the patient developed intermittent complete heart block and an initial bifascicular block which involved an RBBB, which is usually more common in CS than a left bundle branch block [4].

Cardiomyopathy, as evidenced by left ventricular dysfunction, is thought to be secondary to conduction abnormalities, extensive myocardial involvement, or both [3]. In our case, the presence of conduction abnormalities and infiltrative lesions seen on CMR could be the cause of left ventricular dysfunction. Further, we ruled out any ischemic etiologies after a workup with cardiac catheterization showing no significant epicardial disease and after ruling out a hemodynamically significant anomalous RCA.

Elevated cardiac enzymes may indicate disease activity in CS and may be utilized for monitoring [5]. As this finding indicates myocardial injury, prompt evaluation of ongoing ischemia should be warranted. The exact reason for the elevation of cardiac enzymes is unclear and is thought to be secondary to the ongoing inflammatory process in active CS. In this case, due to the significantly elevated troponin I, an ischemic evaluation was done which showed no evidence of significant epicardial disease. This prompted investigation for an infiltrative process that could present as conduction abnormalities and left ventricular dysfunction. 

Other differential diagnoses that should be carefully distinguished from CS include other conditions presenting similarly such as myocarditis and pericarditis. Myocarditis and pericarditis have a variety of causes including viral etiology such as adenovirus and coxsackieviruses [6]. Viral myocarditis and pericarditis usually present acutely and are heralded by a viral prodrome. In addition, Lyme carditis is another possibility and can also present with heart blocks; however, constitutional symptoms and rashes would typically be present [7]. Given the gradual progression of our patient’s presentation and lack of prodromal symptoms, the consideration for infiltrative cardiomyopathy was more likely.

Two major guidelines exist for the diagnosis of CS, namely, the 2014 Heart Rhythm Society (HRS) document and the 2019 Japanese Circulation Society (JCS) criteria. According to the HRS, two diagnostic pathways are available: (a) histological evidence of non-caseating granulomas in the myocardium, and (b) clinical diagnosis [8]. Similarly, the JCS criteria that were recently updated in 2019 had several significant changes including the clinical criteria for clinically diagnosing CS [9]. Our patient satisfied the clinical diagnosis of CS based on new-onset conduction abnormalities and left ventricular dysfunction as well as typical imaging findings on CMR and PET scans.

Although endomyocardial biopsy (EMB) is the gold standard for the diagnosis of CS, the diagnostic yield has been historically low [10]. Without EMB, the constellation of evidence including the presence of complete heart block, unexplained left ventricular systolic dysfunction that is responsive to steroids and immunosuppressive drugs, as well as evidence of LGE in a typical pattern on CMR and FDG PET scan made the diagnosis of CS highly probable in our patient.

Although the cornerstone therapy for treatment is systemic corticosteroids, there is a paucity of data on the optimal initiation, duration, and dosage of immunosuppressive therapies [11,12]. Previous studies failed to prove the efficacy of corticosteroids in reversing conduction abnormalities, preserving left ventricular systolic function, or improving mortality [13]. However, on short-term follow-up, our patient did not have any recurrence of conduction abnormalities after the initiation of steroid therapy. This can possibly be due to an individualized response to treatment; hence, more studies are required to establish appropriate treatment guidelines for patients with CS.

## Conclusions

In summary, CS presents a diagnostic challenge, especially if confounded by evidence of possible ongoing ischemia, and requires a high index of suspicion for diagnosis. Clinical diagnosis is achieved with the constellation of evidence supported by signs and symptoms as well as classic ECG and imaging findings on cardiac MRI/PET-CT, especially when EMB is not feasible. Treatment initiation is, therefore, necessary to achieve control and prevent life-threatening complications such as sudden cardiac death and fatal ventricular arrhythmias.

## References

[1] Rapoport EA, Chidharla A, Mortoti SS (2021). A case of cardiac sarcoidosis with concurrent myocardial ischemia. HeartRhythm Case Rep.

[2] Birnie DH, Nery PB, Ha AC, Beanlands RS (2016). Cardiac sarcoidosis. J Am Coll Cardiol.

[3] Afriyie-Mensah JS, Awindaogo FR, Tagoe EN, Ayetey H (2021). Cardiac sarcoidosis: two case reports. Clin Case Rep.

[4] Garg A, Syed H, Padala SK, Ellenbogen KA, Kron J (2019). Resolution of new left bundle branch block and ventricular tachycardia with immunosuppressive therapy in a patient with cardiac sarcoidosis. HeartRhythm Case Rep.

[5] Birnie DH, Kandolin R, Nery PB, Kupari M (2017). Cardiac manifestations of sarcoidosis: diagnosis and management. Eur Heart J.

[6] Yajima T, Knowlton KU (2009). Viral myocarditis: from the perspective of the virus. Circulation.

[7] Robinson ML, Kobayashi T, Higgins Y, Calkins H, Melia MT (2015). Lyme carditis. Infect Dis Clin North Am.

[8] Birnie DH (2020). Cardiac sarcoidosis. Semin Respir Crit Care Med.

[9] Kawai H, Sarai M, Kato Y (2020). Diagnosis of isolated cardiac sarcoidosis based on new guidelines. ESC Heart Fail.

[10] Terasaki F, Azuma A, Anzai T (2019). JCS 2016 guideline on diagnosis and treatment of cardiac sarcoidosis - digest version. Circ J.

[11] Hulten E, Aslam S, Osborne M, Abbasi S, Bittencourt MS, Blankstein R (2016). Cardiac sarcoidosis-state of the art review. Cardiovasc Diagn Ther.

[12] Ribeiro Neto ML, Jellis CL, Joyce E, Callahan TD, Hachamovitch R, Culver DA (2019). Update in cardiac sarcoidosis. Ann Am Thorac Soc.

[13] Tan JL, Fong HK, Birati EY, Han Y (2019). Cardiac sarcoidosis. Am J Cardiol.

